# Dysregulated MicroRNA Expression Profiles and Potential Cellular, Circulating and Polymorphic Biomarkers in Non-Hodgkin Lymphoma

**DOI:** 10.3390/genes7120130

**Published:** 2016-12-17

**Authors:** Gabrielle Bradshaw, Heidi G. Sutherland, Larisa M. Haupt, Lyn R. Griffiths

**Affiliations:** Genomics Research Centre, Institute of Health and Biomedical Innovation, School of Biomedical Sciences, Queensland University of Technology, 60 Musk Avenue, Kelvin Grove, QLD 4059, Australia; gabrielle.bradshaw@hdr.qut.edu.au (G.B.); heidi.sutherland@qut.edu.au (H.G.S.); larisa.haupt@qut.edu.au (L.M.H.)

**Keywords:** B-cell lymphoma, lymphoid neoplasia, haematological malignancies, micro-RNA (miRNA/miR), circulating miRNA, miRSNP (micro-RNA single nucleotide polymorphism), biomarker

## Abstract

A large number of studies have focused on identifying molecular biomarkers, including microRNAs (miRNAs) to aid in the diagnosis and prognosis of the most common subtypes of non-Hodgkin lymphoma (NHL), Diffuse Large B-cell Lymphoma and Follicular Lymphoma. NHL is difficult to diagnose and treat with many cases becoming resistant to chemotherapy, hence the need to identify improved biomarkers to aid in both diagnosis and treatment modalities. This review summarises more recent research on the dysregulated miRNA expression profiles found in NHL, as well as the regulatory role and biomarker potential of cellular and circulating miRNAs found in tissue and serum, respectively. In addition, the emerging field of research focusing on miRNA single nucleotide polymorphisms (miRSNPs) in genes of the miRNA biogenesis pathway, in miRNA genes themselves, and in their target sites may provide new insights on gene expression changes in these genes. These miRSNPs may impact miRNA networks and have been shown to play a role in a host of different cancer types including haematological malignancies. With respect to NHL, a number of SNPs in miRNA-binding sites in target genes have been shown to be associated with overall survival.

## 1. Introduction

Non-Hodgkin lymphoma (NHL) is biologically distinct from classical Hodgkin lymphoma and comprises a heterogeneous group of lymphoid malignancies involving uncontrolled proliferation of B-, T- and natural killer lymphocytes. Global incidence rates of NHL have been steadily increasing with the highest incidence occurring in developed regions, such as North America, Europe and Australasia [1]. There are over 50 subtypes of NHL, the most common being Diffuse Large B-cell Lymphoma (DLBCL) and Follicular B-cell Lymphoma (FL) [1]. NHL is the fifth most common cancer worldwide, with incidence trends occurring more in older white men, over the age of 70 years; however trends in young men are in line with the human immunodeficiency virus (HIV) epidemic [1]. Rates of DLBCL increase dramatically by the age of 50 years and rates of FL increase from 30 to 70 years [2]. The difference in incidence between racial groups is possibly due to ethnic variations in genes that are involved in regulating the immune system [3]. In the United States in 2012, overall incidence rates of lymphoma were 51% higher in males than females, with occupational exposures during industrial or farm work being the most likely reason for these gender differences, although there is no male dominance in incidence in FL cases [2]. The over-proliferation of lymphocytes may be triggered by a wide range of factors, including exposure to hazardous chemicals, immunosuppressive therapy, immunodeficiency disorders, infection with HIV and Epstein-Barr virus (EBV), heritable genetic risk factors and chromosomal translocations [1,4,5]. DLBCL is diagnosed more frequently than any other lymphoma in adults (up to 40% of cases) and is the most aggressive form of NHL [6]. It is complex to diagnose and treat due to its clinical, pathological and molecular diversity and is considered to consist of different disease entities requiring different forms of treatment [6]. Early gene expression and immunohistochemistry profiling studies have identified three main subgroups of DLBCL: germinal centre B-cell like DLBCL (GCB-DLBCL), activated B-cell-like DLBCL (ABC-DLBCL), and also primary mediastinal DLBCL (PMBL) [7,8,9]. GCB-DLBCL cases have a much better prognosis and survival rate than those with ABC-DLBCL [9]. DLBCL can present “de novo” or as a high-grade transformation from an already diagnosed case of late stage FL [10]. 3-5% of B-cell chronic lymphocytic leukaemia (B-CLL) cases can also transform into DLBCL, called Richter Syndrome [11,12]. FL cases are more indolent than DLBCL and are the second most common of the B-cell tumours (30% of cases) [13]. In FL up to 90% of cases are caused by the t(14;18) translocation, which leads to deregulated expression of the anti-apoptotic B-cell lymphoma 2 (BCL2) protein [14]. As a result of the difficulties in characterisation and management of DLBCL and FL, a number of studies have focused on identifying molecular biomarkers, including microRNAs (miRNAs or MiRs) to aid in their diagnosis and prognosis, and they will be reviewed here.

MiRNAs are short sequences of ribonucleic acid (RNA) that are coded for by miRNA gene sequences of deoxyribonucleic acid (DNA). MiRNAs themselves are non-coding, but are highly complementary to the 3′-untranslated regions (3′ UTRs) of target sequences of transcribed messenger RNA (mRNA) and when a miRNA molecule binds to its specific target it can either inhibit protein translation or cause degradation of the mRNA molecule [15]. In this way, miRNAs act as regulatory molecules and play a role in the negative posttranscriptional regulation of gene expression. A single miRNA is able to target several mRNAs with near perfect base pairing of the 5′ miRNA seed region to its mRNA target, and a single mRNA can be targeted by various miRNAs [16]. The last decade has produced extensive research on miRNAs in cancer with these small, non-coding molecules shown to modulate important cellular processes such as cell growth, proliferation, differentiation, survival and apoptosis [16]. As they are often located at fragile sites and genomic regions on chromosomes that are associated with cancer, they are now studied in almost all cancer types and have been shown to be able to function as either oncogenes or tumour suppressors, and so play a critical role in tumour development [17,18].

Recent advancement in small RNA deep sequencing technology has now enabled the identification of over 2500 mature human miRNAs, according to the latest version of the miRNA database in 2014, miRBase v21 (http://www.mirbase.org/) [19]. The processing pathway and biogenesis of miRNAs has been well characterised in other reviews [20,21] and will not be discussed here.

The majority of mature miRNAs are processed in the cytoplasm and function intracellularly, however some have been found to occur in extracellular circulating environments, such as serum and plasma [22]. Several studies have used the quantitative reverse transcription polymerase chain reaction (qRT-PCR) method to quantitate the levels of miRNA in plasma or serum from cancer patients, to show their use as a potential diagnostic biomarker or tool for predicting disease response [23,24,25]. Chakraborty et al. [26] have recently reviewed cell-free and circulating miRNAs as a diagnostic tool for different cancer types, and they neatly summarise how extracellular miRNAs are secreted through three different pathways, i.e., enclosed within an exosome, enclosed within an apoptotic body and enclosed within a shedding vesicle. Tumour-specific miRNAs in the circulation may be the result of dying or lysed tumour cells releasing exosomes containing miRNAs [27], however there is also evidence of an active release of exosomes containing miR-222 from melanoma tumour cells [28]. Circulating miRNAs are therefore highly stable molecules as they have external protection from RNAse activity with reproducible levels in tumours and biofluids. They provide specific expression patterns or “fingerprints” for different diseases and show potential as a novel type of non-invasive tumour biomarker for cancer diagnosis and prognosis [22].

Single nucleotide polymorphisms or SNPs are single base variations in the DNA sequence. If they result in a codon that codes for an alternate amino acid in the protein sequence they are referred to as missense mutations and may affect protein function, however the majority fall in non-coding regions of the genome and if they have an effect it is most likely to be on gene regulation. Increasing evidence has shown that SNPs in the 3′ UTRs of target mRNAs may affect complementary binding of multiple mature miRNA sequences and thus result in numerous functional consequences including aberrant gene expression and development of tumours [20]. SNPs that affect miRNA expression could also potentially be located in genes in the miRNA biogenesis pathway, as well as in the miRNA genes themselves. Many researchers are now starting to investigate these SNPs and their functional role in disease susceptibility and outcome. To date, several miRSNPs have been identified in haematological malignancies with only a few so far identified in lymphoma [29].

## 2. Dysregulated miRNA Expression Profiles in NHL

B-cell NHL is comprised of a number of different subtypes, including DLBCL, FL, Mantle Cell Lymphoma (MCL), Burkitt Lymphoma (BL), Mucosa-Associated Lymphoid Tissue (MALT), and Marginal Zone Lymphoma (MZL). Since the discovery of miRNAs and their role in tumorigenesis, researchers have aimed to quantitate levels of miRNAs and determine their effect on gene expression in the different subtypes of NHL, focusing mainly on DLBCL and FL.

Evidence for the role of miRNAs in B-cell development and function came in 2007 when Xiao et al. [30] published in Cell that miR-150 controls expression of the c-Myb transcription factor in vivo. Metzler et al. [31] published a ground-breaking finding showing that the expression of pre-miR-155 and its coding gene, *BIC*, was greatly increased in children with Burkitt Lymphoma but not in other paediatric leukaemic samples. This data implied that other molecular factors apart from chromosomal translocations might be involved in the development of lymphomas and opened the door for investigation of expression of a wider selection of miRNAs in B-cell tumours. Lawrie et al. [32] was one of the first groups to perform microarray analysis and qRT-PCR using cell lines to distinguish between DLBCL subtypes, GCB-DLBCL and ABC-DLBCL. They first showed that miR-155, miR-21 and miR-221 were expressed at higher levels in ABC-type than GCB-type cell lines, and that these miRNAs were over-expressed in DLBCL and FL tumour tissues compared to healthy B lymphocytes. Subtypes of DLBCL cases were also analysed and, consistent with expression profiles in the cell lines, these miRNA levels were also increased in the ABC-type cells compared to GCB-type cells. Roehle et al. [6] analysed the expression profiles of 157 known miRNAs, including those already shown to be involved in haematopoiesis (miR-150 and miR-155) and DLBCL and FL (miR-155, miR-210, miR-106a, miR-149, miR-139). They failed to replicate the findings of Lawrie et al. [32] as they had variable reproducibility of immunohistochemical staining which was used to classify DLBCL subtypes, however they were able to show differential expression profiles of 4 miRNAs to distinguish between DLBCL and FL (miR-330, miR-17-5p, miR-106a, miR-210) with an overall accuracy of 98% (109 out of 111 cases). A 9 series miRNA signature was later shown to differentiate ABC-DLBCL and GCB-DLBCL using miRNA profiling and several well-characterised lymphoma cell lines where dysregulated miRNAs were validated by qRT-PCR [33]. All nine miRNAs were expressed at a higher level in ABC- than GCB-like cell lines (miR-17, miR-19b, miR-20a, miR-29a, miR-92a, miR-106a, miR-720, miR-1260, miR-1280). These include 4 members of the miR-17-92 cluster (miR-17, miR-19b, miR-20a and miR-92a), which is a set of miRNAs known to be upregulated with oncogenic potential in lymphoid malignancies [34]. Four miRNAs were shown to be differentially expressed between DLBCL and FL cell lines and normal CD19+ controls (miR-20b, miR-26a, miR-92b and miR-487b) which were not identified in the previous studies of Lawrie et al. [32] and Roehle et al. [6]. It should be noted that this study did not use clinical biopsy samples, only cell lines, which further highlights the molecular heterogeneity between DLBCL and FL immunophenotypes of cell lines and patient tissues. Fassina et al. [35] aimed to differentiate between clinical de novo GCB-DLBCL and non-transforming high grade (grade 3) FL cases using a signature comprising all members of the miR-17-92 cluster (miR-18b, miR-19b, miR-20a, miR-92, miR-93 and miR-106a) and two control miRNAs (miR-150 and miR-210). These were measured by qRT-PCR and all were found to be significantly over-expressed in GCB-DLBCL compared to high grade FL and allowed correct identification of 97% (35/36) GCB-DLBCL cases. A more comprehensive investigation of miRNA expression in DLBCL and FL was performed by Lawrie et al. [10], this time using a microarray platform containing 464 human probes, in order to validate miRNA profiles in clinical material rather than in lymphoma cell lines. Here an unsupervised cluster analysis showed distinct expression patterns and specific miRNA signatures, including members of the miR-17-92 cluster, for DLBCL and FL, and was able to correctly identify the subtype in >95% of cases. They were also interested in comparing miRNA expression patterns between de novo DLBCL cases and FL cases that had undergone transformation, and showed differential expression of 12 miRNAs that predicted >85% of transformed versus de novo DLBCL. 6 miRNAs (miR-223, miR-217, miR-222, miR-221, let-7i and let-7b) were dysregulated and found to predict subsequent transformation from indolent FL to transformed DLBCL. Interestingly, the let-7 family of miRNAs had been shown previously to target *c-myc*, which was associated with transformation of FL to DLBCL [36]. Although the number of FL cases in transformation was small (*n* = 7), these data suggest that these miRNAs may have the potential to identify those FL patients at risk of high-grade transformation.

More recently, global miRNA expression profiling of tumour tissue samples and cell lines identified 27 miRNAs that enabled accurate differentiation between DLBCL and BL, including distinct miRNA signatures for differentiation between the GCB-, ABC- and PMBL subgroups of DLBCL [37]. Their findings correspond to gene expression profile (GEP) data, however GEP methodology has not been well accepted into clinical practice. Iqbal et al. [37] showed miR-155 to be significantly associated with ABC-DLBCL, which was consistent with previous findings [32], while miR-28-3p and miR-28-5p were both upregulated in GCB-DLBCL.

FL cases that lack the t(14;18) translocation and also lack *BCL-2* expression run the risk of being misdiagnosed as benign follicular hyperplasia (FH) [5]. The t(14;18)(q32.3;q21.3) translocation involves juxtaposition of the *BCL-2* oncogene on chromosome 18 to the promoter region of an immunoglobulin heavy chain gene *Ig_H_* on chromosome 14, leading to constitutive over-expression of the anti-apoptotic BCL-2 protein [5]. Leich et al. [13] were the first group to characterise miRNA profiles in t(14;18)-negative cases of FL. They were able to show a distinct miRNA expression profile for 17 miRNAs in support of a late germinal centre B-cell phenotype, which could potentially give these tumour cells a propensity for proliferation. Down-regulation of 5 miRNAs (miR-16, miR-26a, miR-101, miR-29c and miR-138) in the t(14;18)-negative FL cases was associated with significantly increased expression of potential target genes, such as the miR-16 target *CHECK1*, known to be involved in cell cycle regulation, apoptosis and B-cell differentiation [13]. MiR-138 was also previously identified to be differentially expressed between GCB- and ABC immunophenotypes of DLBCL de novo cases, suggesting a partial loss of the germinal centre B-cell phenotype in t(14;18)-negative FLs [10]. As miRNAs were not yet well characterised in FL by 2012, another group analysed the miRNA profiles of B-cell enriched FL tumour cells and FH cells from 16 patients by assaying 851 human miRNAs [38]. They showed differential expression of 133 miRNAs between FL and FH, where 44 miRNAs in three groups generated a unique signature for FL diagnosis. A NanoString nCounter assay identified tandem gene expression profiles for predicting miRNA targets, where functional studies identified genes involved in cell proliferation and tumour response to chemotherapy, such as the miR-20a/-20b target *CDKN1A/p21* and the miR-194 target *SOCS2* [38]. The levels of these three miRNAs were increased while their target genes had decreased expression, indicating a potential role in lymphomagenesis and survival. According to Akasaka et al. [39], presence of a *BCL-6* rearrangement may predispose transformation from FL to DLBCL. Two FL subtypes have been classified, e.g., FL with *BCL-6* locus rearrangement with or without *BCL-2* expression (FL^BCL2+/BCL6+^, FL^BCL2−/BCL6+^) and FL with diffuse growth (DFL) [40]. More recently Gebauer et al. aimed to identify miRNA signatures between the two FL subtypes. They found several miRNAs that were differentially expressed between FL^BCL2+/BCL6+^ and FL^BCL2−/BCL6+^, and also DFL, with up-regulation of oncogenic miRNAs and down-regulation of tumour suppressor miRNAs [40]. Cluster analysis, however, showed no miRNA signatures distinct from the reference group, that is typical FL^BCL2/BCL6−^, for either subtype. These data suggest an involvement of miRNAs in the pathogenesis of FL and its subtypes, with deregulated miRNAs corresponding to an increased risk of transformation and an inferior prognosis.

A summary of these studies on dysregulated miRNAs and their potential biomarker function in NHL can be found in Table 1.

## 3. Functionally Significant Cellular miRNAs in NHL

Studies previously mentioned have focused mainly on the quantification of miRNA levels to distinguish between DLBCL, FL and their subtypes, and have shown their potential to behave as molecular biomarkers for diagnosis and prognosis. An aberrant miRNA signature is useful, however it is important to be able to understand the functional role of these miRNAs and how they regulate expression of their target genes, to enable clinicians to determine different therapeutic modalities for individual patients as well as response to treatment and resistance to chemotherapy. Advancement in the treatment of DLBCL includes a combination of rituximab (monoclonal antibody) with cyclophosphamide, vincristine, doxorubicin and prednisolone (R-CHOP), which has resulted in a large increase in overall survival rates, however up to 33% of patients will still eventually succumb to this disease [41,42]. Studies investigating functional diagnostic as well as prognostic biomarkers in clinical tumour tissue samples are therefore required.

Two studies have used targetome profiling to implicate specific miRNAs in lymphomagenesis [43,44]. A ribonucleoprotein immunoprecipitation-microarray (RIP-Chip)-based approach was used in HeLa cell lines to identify members of the miR-202 and miR-618 targetome, that is, their range of target genes. MiR-618 dysregulation has been associated with liver cancer [45] and male breast cancer [46].

MiR-202 dysregulation has been associated with breast cancer [47] and was decreased in FL cells compared to FH cells [38] implicating a potential tumour suppressor role in lymphoma. 128 miR-618 and 141 miR-202 potential targets were identified which suggested involvement in pathways related to haematologic function and lymphoma, however targets should still be verified in lymphoma samples.

Wang et al. [48] first screened miRNA expression profiles in DLBCL tissues and cell lines and then performed target gene expression analysis to identify miRNAs that could be associated with lymphoma proliferation and invasion. MiR-144 was significantly decreased in samples and cell lines and was negatively correlated with *BCL-6* expression. They showed for the first time that miR-144 directly targets the *BCL-6* gene. MiR-144 has been identified as a tumour suppressor in osteosarcoma [49] and hepatocellular carcinoma [50]. The BCL family of proteins can have an anti-apoptotic function in haematological malignancies and hence miR-144 appears to be a viable biomarker and therapeutic target for lymphomagenesis [48]. It would be of interest to identify variants in the miR-144 and other *BCL-6*-associated miRNAs′ precursor sequences, as well as variants in the *BCL-6* target gene to perform genetic association analysis in DLBCL and also FL cases.

MiR-21 was identified as an independent prognostic marker in de novo cases of DLBCL as high miR-21 expression was associated with a longer relapse-free survival using Kaplan-Meier survival analysis (*p* < 0.05) [32] and miR-21 inhibition in HeLa cells caused increased proliferation [51]. This data does not seem to be in line with a functional analysis of miR-21 target genes which showed that miR-21 directly targets *PTEN*, which when down-regulated will allow activation of protein kinase B resulting in significant anti-apoptotic effects in tumour cells [52]. More recently, miR-21 levels were shown to be overexpressed in PBMCs from B-NHL patients and significantly higher in patients in stages III/IV than I/II [53]. PTEN expression was significantly lower in patients, compared to healthy controls, which would be expected to have an anti-apoptotic effect and shorten overall survival periods. MiR-21 levels dropped to normal in patients in complete remission and therefore improved outcome was indicated by a negative association of miR-21 expression and post-chemotherapy survival rates of these patients.

Other miRNAs known to play a role in cellular proliferation and apoptosis, such as miR-224 and miR-34a, have been analysed for prognosis and clinical outcome. 258 DLBCL patients undergoing R-CHOP therapy were investigated for levels of miR-224 expression, which was shown to be deregulated in other cancers [58]. MiR-224 expression was significantly lower in the DLBCL biopsy samples (*n* = 258) than in normal lymphoid tissues (*n* = 40) with no significant difference in expression between GCB-DLBCL and ABC-DLBCL subgroups. The authors also showed that patients with a higher miR-224 expression level achieved an improved rate of complete remission compared with the group expressing lower miR-224 levels [59]. MiR-34a expression was significantly lower in MALT and DLBCL lymphoma tissues when compared to healthy lymphocytes and inversely correlated with high expression of its target genes *FOXP1*, *p53* and *BCL-2* in stage III and IV. This was associated with an unfavourable outcome in both lymphoma subtypes but a significantly lower overall survival in DLBCL [60]. These changes in expression are therefore potential prognostic biomarkers for use in clinical practice. It has been shown most recently using in vitro and in vivo studies that increased levels of miR-34a were observed in cell lines sensitive to doxorubicin, a drug in the R-CHOP regime, and this high expression had a prognostic impact on improved overall survival [61]. Downregulation of *FOXP1*, a known target of miR-34a, could therefore potentially sensitise tumour cells to doxorubicin and improve efficacy of the drug in chemo-resistant patients.

Iqbal et al. [37] investigated miRNA association with resistance to R-CHOP therapy and showed miR-155 to be significantly correlated with therapy failure. As they had previously shown increased miR-155 levels in ABC-DLBCL and downregulation of genes regulating growth factor signalling and cell cycle, they determined therapy failure may possibly be the result of activation of the PI3K-AKT signalling pathway [62]. Interestingly, high levels of miR-155 have been found in breast cancer cases experiencing chemo-resistance [63]. This highlights the AKT pathway as a potential therapeutic target for overcoming drug resistance in ABC-DLBCL patients.

## 4. Circulating miRNAs in NHL

MiRNAs are detectable outside of cells in blood serum and/or plasma (circulating miRNAs) and have the potential to function as non-invasive diagnostic and prognostic markers in cancer [26,64]. Methods used for profiling circulating miRNAs are the same as those for cellular miRNAs, that is, qRT-PCR, microarray and next-generation or deep sequencing [26]. Only a few studies have attempted to investigate circulating miRNA profiles in patients with NHL with promising results for diagnosis, subtype classification, prognosis and response to R-CHOP treatment. Lawrie et al. [23] were the first to publish their discovery of circulating miRNAs in biological fluids in any disease. They used qRT-PCR to detect miRNAs in the serum of DLBCL patients and showed differential expression of these miRNAs in patients and healthy controls. Similar to their previous study investigating differential expression of miRNAs in tumour cells [32], they were able to show increased expression of tumour-associated miRNAs (miR-155, miR-210 and miR-21) in the serum of DLBCL patients compared to control sera samples [23]. They showed that increased serum miR-21 expression was significantly associated with relapse-free survival in these patients, which is in keeping with their findings on the prognostic value of miR-21 in de novo DLBCL cases [32], however they did not directly correlate miR-21 levels in tumour cells and serum in the same cohort of patients. A follow up study by Chen et al. [55] confirmed these findings in a Chinese population and showed that not only was miR-21 expression higher in ABC-DLBCL serum compared to GCB-DLBCL serum, but the circulating levels of miR-21 in sera from DLBCL patients was significantly associated with matched tumour tissue samples (*p* = 0.0001). Furthermore, miR-21 upregulation was higher in stages I/II compared to III/IV, which contradicts later findings in PBMCs by Sun et al. [53] mentioned earlier, and significantly associated with improved prognosis. This discrepancy could be due to alternative miR-21 sources being used in different studies, or it could mean there are other miR-21 targets influencing apoptosis or cellular proliferation that have not yet been discovered. Altogether these findings highlight the potential of serum miR-21 to function as a diagnostic and prognostic non-invasive biomarker for DLBCL and its subtypes, however studies should be validated in other larger populations.

Another group measured levels of 7 miRNAs in serum of DLBCL patients compared to healthy controls using qRT-PCR and showed 4 miRNAs to be significantly increased in DLBCL (miR-15a, miR-16-1, miR-29c, miR-155) while miR-34a was decreased [24]. These data could serve as the basis for further functional studies in larger populations as there are limitations to this study, such as the small sample size (*n* = 75) and measuring low levels of miRNAs with the lack of standard curve calibration in their qRT-PCR assay.

More recently Yuan et al. [57] aimed to investigate the correlation, if any, between circulating miRNAs and chemoresistance in DLBCL patients. At first they showed dysregulated expression of eight miRNAs in serum from 56 DLBCL patients prior to treatment compared to healthy controls: miR-155, miR-200c, miR-130a, miR-125b and miR-21 were upregulated, whereas miR-29c, miR-451 and miR-145 were downregulated. They also showed a significant association of these miRNAs between serum and matching tumour biopsy samples. Out of the 56 patients, 21 experienced resistance to R-CHOP, and 35 were sensitive to the therapy. The group found that only miR-130a and miR-125b expression was significantly upregulated in the drug resistant group compared to the drug sensitive group and in addition, high levels of miR-130a and miR-125b were associated with a worse prognosis using Kaplan-Meier survival analysis curves, however this was only significant for miR-125b, as miR-130a was not significant after Multivariate Cox regression analysis [57]. Another study showed a correlation between miR-21 knock-down in a DLBCL cell line and decreased resistance to CHOP treatment [65], however Yuan et al. [57] were not able to show that increased serum miR-21 levels increased resistance to R-CHOP in their study.

Serum miRNA levels are evidently promising non-invasive biomarkers of DLBCL, however further studies are required for validation and elucidation of molecular mechanisms of serum miRNAs in drug resistance and prognosis. No studies to date have investigated serum miRNA levels in FL or other less common NHL subtypes. As bone marrow involvement can occur in 50% of grade 1 FL cases [66], one study has measured miRNA levels in bone marrow smears from FL patients, and identified an aberrantly expressed miRNA profile between FL patients and normal controls using a TaqMan real time PCR miRNA array method [56]. 39 miRNAs were significantly decreased, of which miR-451 was the most decreased, and 27 miRNAs were significantly increased, of which miR-338-5p showed the highest increase. The authors claim these miRNAs may serve as biomarkers to predict the invasion of FL cells into the bone marrow without having to perform a biopsy, and that bone marrow smear samples could serve as another reliable source of miRNAs. A summary of these studies on circulating miRNAs can also be found in Table 1.

## 5. MiRNA-Related SNPs in NHL

Genetic variation in microRNA regulatory pathways in cancer are now being investigated in depth. These polymorphisms can occur in miRNA biogenesis pathway genes, in miRNA genes in different regions, i.e., pre-miRNA, seed or mature sequences, and in miRNA-binding site target genes. These polymorphisms may alter miRNA function as either an oncogene or tumour suppressor and potentially behave as diagnostic and prognostic biomarkers for clinical use. These miRNA variants or SNPs (miRSNPs) have been extensively reviewed in a range of cancer types [20,67,68], including haematological malignancies [29], however no reviews to date have summarised the new research on these miRSNPs in NHL specifically. Currently the significant miRSNPs identified in NHL are very few with even fewer functional studies to back them up and have only been observed in particular subtypes, although in some studies there has been no stratification of subtypes and all patients are classified as NHL.

The first report of an association between a heritable polymorphism at a miRNA locus and lymphoma was in 2012 when Schuetz et al. [69] found a variant near miR-155 (rs928883) using Sanger sequencing, and found it to be significantly associated with risk of MZL in Caucasians of European ancestry after correction for multiple testing (*p* = 0.0272). Functional analysis was not carried out for this SNP. Four mutations in the mature miR-142-5p/-3p sequences, and three in the miR-142 precursor sequence, were identified by Kwanhian et al. [70] in the same year, who also claimed to have made the first report describing mutations in a miRNA gene in a lymphoma subtype, DLBCL. Their functional analysis showed a potential loss of function for these mutations and loss of responsiveness of the known miR-142-3p targets *RAC1* and *ADCY9*.

Two polymorphisms were identified in the ethnic Han Chinese, that is, miR-146a rs2910164 and miR-196-a2 rs11614913, which were both significantly associated with an increased risk of DLBCL and NHL, respectively [71,72]. The rs11614913 T > C variant is located in the miR-196-a2 gene and was shown by Hoffman et al. [17] to possibly play an oncogenic role in breast cancer by leading to less efficient processing of the miRNA precursor and reduced capacity of the mature form to influence its targets. Li et al. [72] performed a genotype-phenotype correlation analysis on NHL tissue samples (*n* = 59) and confirmed that the rs11614913 TC/CC genotype affects levels of mature miR-196-a2 expression with an increase in expression in carriers of the variant C allele. Furthermore, miR-196-a2 rs11614913 was inversely associated with NHL susceptibility in Caucasian participants only in an AIDS-NHL cohort [73]. The rs2910164 G > C variant is located in the stem structure of the miR-146a precursor sequence. Zhuang et al. [71] showed that miR-146a expression was significantly upregulated in DLBCL patients (*n* = 56) compared to controls (*n* = 28) and the polymorphism affects levels of mature miR-146a expression with a significant decrease in expression in patients with the CC genotype. Interestingly, we had shown a genetic association between miR-146a rs2910164 (G > C) and increased susceptibility to sporadic breast cancer in an Australian case-control cohort [74]. Validation of these findings is required in other larger DLBCL populations of different ethnicities and further functional analyses should be carried out.

MiR-618 and miR-202 were found to play a direct role in follicular lymphomagenesis with variants in their precursor sequences showing a significant association with FL by influencing levels of target gene expression. The pre-miR-618 SNP rs2682818 and pre-miR-202 SNP rs12355840 were genotyped in a population-based case-control study of 455 NHL patients and 527 controls where individuals with one or both of the variant alleles were at a higher risk for FL [43,44]. Vectors were used to test the functional effect of the variant and wild type alleles on miR-618 and miR-202 expression and were transfected into HeLa cells, after which levels of primary and mature miRNA transcripts were measured by qPCR. They were able to show that presence of the variant T and G alleles can reduce in vitro production of mature miR-618 and miR-202 levels, respectively. Due to a lack of patient RNA samples, however, they were not able to directly correlate their genotyping data with miRNA gene expression [43,44]. These studies are some of the first to investigate the functional tumour suppressor role of miRSNPs in lymphomagenesis, however expression levels of miR-618 and miR-202 and their identified targets require examination in clinical samples.

One study looked at SNPs located in the miRNA processing genes to determine an association with NHL. An independent association was found after multivariate analysis between rs197412 located in the coding region of *GEMIN3* (together with RISC, *GEMIN3* will aid in selection of one strand of the miRNA duplex as the mature miRNA and guide it to its target) and overall survival in NHL patients (*p* = 0.013) which also carried over to the DLBCL and T-cell lymphoma subtypes in their cohort [75]. Furthermore, this SNP has also been associated with AIDS-NHL susceptibility with evidence to show that variation in this gene may affect miR-222 expression [73].

Polymorphisms located in the 3′ UTRs of miRNA target genes may affect the precision complementarity and binding affinity between miRNA binding sites and their target mRNAs and therefore disrupt gene expression and protein levels [29]. These SNPs occur more frequently in the genome affecting only target gene expression and its downstream pathway effectors leading to a limited range of effects [68]. A few studies have identified SNPs in 3’ UTR targets that disrupt miRNA binding sites and have been shown to have an effect on gene expression and clinical significance in cancer patients [76,77,78]. A number of these SNPs have also been identified in individual studies in NHL and associated with disease prognosis. Li et al. [79] identified multiple variants by sequencing the 3′ UTR of the *TP53* gene in DLBCL patients and confirmed that they have prognostic significance. Three novel single nucleotide variants were shown to disrupt miR-125b binding to their *TP53* target causing upregulation of p53 expression and an increase in apoptosis, while in contrast the rs78378222 CC SNP located in the polyadenylation signal (PAS) and not a putative miRNA binding site, was shown to reduce p53 expression and subsequently reduced apoptosis. Three studies on Han Chinese NHL patients were performed by collaborating authors on known SNPs in 3′ UTRs of miRNA targets in order to investigate an association between these SNPs and disease outcome [80,81,82]. The first study investigated four SNPs and showed the rs3660 SNP GG genotype in the *KRT81*-miR-17 interaction was significantly associated with poorer outcome (*p* = 0.003) after multivariate analysis [80]. Similarly, this SNP was previously associated with tumour recurrence and worse overall survival in multiple myeloma [83]. The second study evaluated the effect of a polymorphism in the *SET8* 3′ UTR and showed the rs16917496 CC genotype of the SNP in the miR-502 binding site was significantly associated with increased survival (*p* = 0.041) after multivariate analysis [82] and may have prognostic value in a clinical setting. Thirdly, an analysis was performed on another four SNPs, however the disrupted miRNA binding sites were not specified. The rs4901706 AA genotype of the *C14orf101* 3′ UTR was significantly associated with a longer overall survival (*p* = 0.015) and was identified as an independent predictor of overall survival (*p* = 0.033) by multivariate analysis [81]. None of these studies, however, showed any significant associations between these and other miRNA binding site SNPs and risk of NHL in their case-control cohorts. Peckham et al. [73] found an increased risk for systemic HIV/AIDS NHL compared to HIV positive controls with the variant allele of the *HIF1A* 3′ UTR SNP rs2057482 located within a miRNA-196a binding site.

The results from these studies are preliminary and the underlying mechanisms behind how these SNPs influence risk and survival in NHL remain unclear. Therefore validation in other populations and functional in vitro experiments in clinical tumour samples should be performed to confirm and elucidate these findings. A summary of these studies can be found in Table 2 and Table 3.

## 6. Challenges and Future Developments

DLBCL is an aggressive cancer with up to 40% of patients unable to achieve relapse-free survival with primary therapies. Studies that set out to investigate methods that enable early diagnosis and subtype classification as well as prediction of drug resistance and disease outcome for individual patients are of paramount importance. Over the last decade researchers have identified distinct miRNA profiles for the diagnosis and prognosis of the common subtypes of NHL, and some have gone on to further investigate the regulatory roles these miRNAs play in lymphomagenesis, enabling improvements in clinical management. 

Methodologies used to measure miRNA levels in tumour biopsies and biofluids include qRT-PCR, microarray and next generation sequencing (NGS), while deep sequencing of small miRNAs (miRNA-seq) has been recently developed to allow the discovery of novel biomarker miRNAs and can be performed on different high-throughput platforms, including Ion Torrent and Illumina. While NGS is more rapid and advanced, there is still a level of bias between microarray and deep sequencing identification and quantification of miRNAs in terms of base composition [84]. As a result, biomarkers discovered through NGS should be carefully considered and properly validated. Using sequencing miRNA profiles for determination of subtype and survival adds prognostic value to the Revised International Prognostic Index (R-IPI) which is equivalent to the gold standard for selecting patients with a poor outcome [85]. MiRNA-seq was used in a recent study to profile DLBCL patient samples and perform an integrated analysis of the DLBCL miRNA targetome (miRNome) [86]. Lim et al. [86] used two cohorts, discovery and validation, in which five known and one unknown novel candidate miRNA (miR-28-5p, miR-324-5p, miR-214-5p, miR-339-3p, miR-5586-5p, NOVELM00203M) were associated with patient survival, independently of the IPI scores. The miRNA-seq data was integrated with gene expression data from the same patients which revealed that upregulation of miRNAs in DLBCL can lead to aberrant regulation of genes involved in epigenome modulation, and comparison of patient profiles with miRNA-seq libraries of other diseases identified miRNAs (including three novel candidates) that were specifically involved with B-cell biology, suggesting these miRNAs may be involved in lymphomagenesis [86].

Cammaerts et al. [87] have developed a software tool for predicting the impact of gene variants on miRNA expression, called miRVas. This was designed to overcome time-consuming processes and manual errors experienced with determining secondary miRNA structures with variants using the miRSNP database tool, especially when more than one variant is investigated. Also, for novel variants, databases cannot be used to obtain variant information. Hence, the miRVas tool is more user-friendly and can speed up and facilitate evaluation of the impact of several genetic variants on miRNAs simultaneously. As functional analysis of miRSNPs is costly and takes time, and with the availability of increasing sequencing driven identification of variants in or near miRNA genes, it is necessary to accurately predict a functional effect to enable selection of the most appropriate candidates for further analysis.

The biggest challenge with qRT-PCR or microarray miRNA profiling of biofluids is the lack of a suitable normalisation control or internal reference miRNA. Endogenous controls (ECs) such as small nucleolar RNAs (snoRNAs) have been validated for use in serum and plasma samples [88]. It was found that U6 is unsuitable as an EC, while miR-16 and miR-24 have stable levels of expression and are less likely to degrade after repeat freeze-thaw cycles. According to Mestdagh et al. [89], it is possible that mean miRNA expression levels can reduce variation between samples and more accurately detect aberrant expression profiles rather than using ECs. Although NGS is more specific and able to detect novel mature miRNA isoforms (isomiRs), it may not detect lower levels of miRNAs in biofluids, and so qRT-PCR or Nanostring nCounter assays are perhaps more feasible for serum and plasma profiling [90].

## 7. Conclusions

The ability of miRNAs to behave as molecular biomarkers for diagnosis, subtype classification, drug resistance and prognosis in NHL is widely apparent. With advancing deep sequencing technologies the identification of novel miRNA biomarkers is underway, however in order to properly elucidate the roles of cellular, circulating and polymorphic miRNAs in lymphocyte biology and pathophysiology of NHL, they need to be validated in other larger populations and functional studies need to be performed in viable clinical samples, such as formalin-fixed paraffin-embedded (FFPE) tumour tissue biopsies, which are suitable for reliable miRNA detection and measurement for more than eight years from the time of processing [32]. Although results for circulating miRNAs are very new, their ability to be used as non-invasive biomarkers for patients undergoing R-CHOP therapy for ongoing detection of response to treatment and relapse is highly promising.

## Figures and Tables

**Table 1:** A summary and comparison of dysregulated miRNAs that could be used as potential diagnostic, subtype and prognostic biomarkers in non-Hodgkin lymphoma (NHL).

miRNA (New Annotation [54])	Source	Expression	B-NHL Subtype	Biomarker	Reference
**miR-155 (hsa-mir-155-5p), miR-21 (hsa-mir-21-5p), miR-221 (hsa-mir-221-3p)**	Cell lines	Increased	ABC-DLBCL vs. GCB-DLBCL	Subtype	Lawrie, 2007 [32]
	Patient cells and biopsy	Increased	DLBCL, FL vs. healthy B cells	Diagnostic	Lawrie, 2007 [32]
	Patient cells and biopsy	Increased	ABC-DLBCL vs. GCB-DLBCL	Subtype	Lawrie, 2007 [32]
**miR-155 (hsa-mir-155-5p), miR-21 (hsa-mir-21-5p), miR-210 (hsa-mir-210-3p)**	Serum	Increased	DLBCL vs. healthy controls	Diagnostic	Lawrie, 2008 [23]
**miR-21 (hsa-mir-21-5p)**	Patient cells	Increased	de novo DLBCL	Prognostic—longer RFS	Lawrie, 2007 [32]
	Serum	Increased	de novo DLBCL	Prognostic—relapse-free survival	Lawrie, 2008 [23]
	Cell lines	Increased	ABC-DLBCL vs. GCB-DLBCL	Subtype	Chen, 2014 [55]
	Serum	Increased	DLBCL vs. normal controls	Diagnostic	Chen, 2014 [55]
	Serum	Increased	ABC- vs. GCB-DLBCL	Subtype	Chen, 2014 [55]
				Prognostic—higher in stages II/III—improved outcome	Chen, 2014 [55]
	PBMCs	Increased	B-NHL	Prognostic—higher in stages III/IV	Sun, 2015 [53]
**miR-330 (hsa-mir-330-3p), miR-17-5p, miR-106a (hsa-mir-106a-5p), miR-210 (hsa-mir-210-3p)**	Patient biopsy	Dysregulated	DLBCL vs. FL	Subtype	Roehle, 2008 [6]
**miR-125b (hsa-mir-125b-5p), miR-143 (hsa-mir-143-3p), miR-451 (hsa-mir-451a), miR-145 (hsa-mir-145-5p)**	Patient cells and biopsy	Increased	DLBCL vs. FL	Subtype	Lawrie, 2009 [10]
**miR-223 (hsa-mir-223-3p), miR-217 (hsa-mir-217), miR-222 (hsa-mir-222-3p), miR-221 (hsa-mir-221-3p), let-7i (hsa-let-7i-5p) and let-7b (hsa-let-7b-5p)**	Patient cells and biopsy	Dysregulated	de novo DLBCL vs. transformed FL	Subtype	Lawrie, 2009 [10]
**miR-17-92 cluster, miR-29a (hsa-mir-29a-3p), miR-106a (hsa-mir-106a-5p), miR-720 (not annotated), miR-1260 (hsa-mir-1260a), miR-1280 (not annotated)**	Cell lines	Increased	ABC-DLBCL vs. GCB-DLBCL	Subtype	Culpin, 2010 [33]
**miR-20b (hsa-mir-20b-5p), miR-26a (hsa-mir-26a-5p), miR-92b (hsa-mir-92b-3p), miR-487b (hsa-mir-487b-3p)**	Cell lines	Increased	DLBCL vs. FL	Subtype	Culpin, 2010 [33]
**miR-17-92 cluster, miR-150 (hsa-mir-150-5p), miR-210 (hsa-mir-210-3p)**	Patient biopsy	Increased	GCB-DLBCL vs. high grade FL	Subtype	Fassina, 2012 [35]
**miR-15a (hsa-mir-15a-5p), miR-16-1 (hsa-mir-16-1-3p), miR-29c (hsa-mir-29c-3p), miR-155 (hsa-mir-155-5p)**	Serum	Increased	DLBCL vs. healthy controls	Diagnostic	Fang, 2012 [24]
**miR-34a (hsa-mir-34a-5p)**	Serum	Decreased	DLBCL vs. healthy controls	Diagnostic	Fang, 2012 [24]
**miR-451 (hsa-mir-451a)**	Bone marrow smear	Decreased	FL vs. normal controls	Diagnostic	Takei, 2014 [56]
**miR-338-5p (hsa-mir-338-5p)**	Bone marrow smear	Increased	FL vs. normal controls	Diagnostic	Takei, 2014 [56]
**miR-17-92 cluster and their paralogues miR-18b (hsa-mir-18b-5p), miR-20b (hsa-mir-20b-5p), miR-106a (hsa-mir-106a-5p)**	Patient biopsy	Increased	BL vs. normal B cells and T-cells	Diagnostic	Iqbal, 2015 [37]
**miR-155 (hsa-mir-155-5p)**	Patient biopsy	Decreased	BL vs. DLBCL	Subtype	Iqbal, 2015 [37]
	Activated B cells and ABC-DLBCL cell lines	Increased	ABC-DLBCL vs. GCB-DLBCL	Subtype	Iqbal, 2015 [37]
	Biopsy from patients treated with R-CHOP	Increased	ABC-DLBCL	Prognostic—treatment failure	Iqbal, 2015 [37]
**miR-155 (hsa-mir-155-5p), miR-200c (hsa-mir-200c-3p), miR-130a (hsa-mir-130a-3p), miR-125b (hsa-mir-125b-5p), miR-21 (hsa-mir-21-5p)**	Serum	Increased	DLBCL vs. healthy controls	Diagnostic	Yuan, 2016 [57]
**miR-451 (hsa-mir-451a), miR-145 (hsa-mir-145-5p)**	Serum	Decreased	DLBCL vs. healthy controls	Diagnostic	Yuan, 2016 [57]
**miR-125b (hsa-mir-125b-5p), miR-130a**	Serum	Increased	DLBCL	Prognostic—poor outcome	Yuan, 2016 [57]

**Table 2:** A summary of single nucleotide polymorphisms in miRNA genes or their processing genes and their association and function in different non-Hodgkin lymphoma (NHL) populations and subtypes.

miRNA Gene/Processing Gene	New Annotation [54]	SNP/Mutation	B-NHL Subtype	Association/Function	Population	Reference
**hsa-miR-155**	hsa-miR-155-5p	rs928883 (A > G)	MZL	Increased risk	Canadian (569 cases vs. 547 controls)	Schuetz, 2012 [69]
**miR-142, miR-142-5p, miR-142-3p**	hsa-miR-142-5p hsa-mir-142-3p		DLBCL	20% of cases are mutated with a loss of function	European (56 cases)	Kwanhian, 2012 [70]
**Pre-miR-202**	hsa-mir-202	rs12355840 (A > G)	FL	Increased risk	American (455 cases vs. 527 controls)	Hoffman, 2013 [43]
**Pre-miR-618**	hsa-mir-618	rs2682818 (G > T)	FL	Increased risk	American (455 cases vs. 527 controls)	Fu, 2014 [44]
**Pre-miR-146a**	hsa-mir-146a	rs2910164 (G > C)	DLBCL	Increased risk	Chinese (280 cases vs. 300 controls)	Zhuang, 2014 [71]
**miR-196a2**	hsa-mir-196a2	rs11614913 (T > C)	NHL	Increased risk	Chinese (318 cases vs. 320 controls)	Li, 2015 [72]
			CNS HIV/AIDS NHL	Decreased risk	American HIV+ men (180 cases vs. 529 controls)	Peckham, 2014 [73]
***GEMIN3***		rs197412 (T > C)	NHL/DLBCL	Independently associated with overall survival	Chinese (168 cases vs. controls)	Gao, 2015 [75]
			HIV/AIDS NHL	Increased risk with increased serum miR-222	American HIV+ men (180 cases vs. 529 controls)	Peckham, 2014 [73]

**Table 3:** A summary of single nucleotide polymorphisms and novel single nucleotide variants in the 3’UTRs of miRNA target genes and their association and function in different non-Hodgkin lymphoma (NHL) populations and subtypes, PAS, polyadenylation signal; SNV, single nucleotide variant.

Target Gene	Disrupted miRNA Binding Site (New Annotation [54])	SNP	B-NHL Subtype	Association/Function	Population	Reference
***TP53* 3′ UTR**	Germline SNP in PAS sequence	rs78378222 (A > C)	DLBCL	Decreased p53 protein expression and reduced apoptosis with CC genotype/ prognostic significance	American (244 cases training set and 247 cases validation set)	Li, 2013 [79]
	miR-125b (hsa-miR-125b-5p)	3 novel SNVs (733U, 737A, 739A)		Negative regulation pf p53 altering p53 levels in vitro		
***KRT81* 3′ UTR**	miR-17 (hsa-miR-17-3p/5p)	rs3660 (G > C)	NHL	Shorter survival time	Chinese (210 cases vs. 233 controls)	Xie, 2014 [80]
***C14orf101* (TMEM260) 3′ UTR**	Not specified	rs4901706 (A > G)	DLBCL	Shorter survival time/independent predictor of overall survival	Chinese (96 cases vs. 90 controls)	Yang, 2014 [81]
***HIF1A* 3′ UTR**	miR-196a (hsa-mir-196-5p)	rs2057482 (C > T)	Systemic HIV/AIDS NHL	Increased risk	American HIV + men (180 cases vs. 529 controls)	Peckham, 2014 [73]
***SET8* 3′ UTR**	miR-502 (hsa-mir-502-5p)	rs16917496 (C > T)	NHL	Shorter survival time (CC vs. CT only)	Chinese (244 cases)	Diao, 2014 [82]
